# Let us to the TWISST; Plan, Simulate, Study and Act

**DOI:** 10.1097/pq9.0000000000000664

**Published:** 2023-07-10

**Authors:** Nora Colman, Kiran B. Hebbar

**Affiliations:** 1 From the Department of Pediatrics, Division of Pediatric Critical Care, Children’s Healthcare of Atlanta, Atlanta, Ga.

## Abstract

Translational Work Integrating Simulation and Systems Testing (TWISST) is a novel application of simulation that augments how we discover, understand, and mitigate errors in our system. TWISST is a diagnostic and interventional tool that couples Simulation-based Clinical Systems Testing with simulation-based training (SbT). TWISST tests environments and work systems to identify latent safety threats (LSTs) and process inefficiencies. In SbT, improvements made to the work system are embedded in hard wire system improvements, ensuring optimal integration into clinical workflow.

**Methods:**

Simulation-based Clinical Systems Testing approach includes simulated scenarios, Summarize, Anchor, Facilitate, Explore, Elicit debriefing, and Failure Mode and Effect Analysis. In iterative Plan-Simulate-Study-Act cycles, frontline teams explored work system inefficiencies, identified LSTs, and tested potential solutions. As a result, system improvements were hardwired through SbT. Finally, we present a case study example of the TWISST application in the Pediatric Emergency Department.

**Results:**

TWISST identified 41 latent conditions. LSTs were related to resource/equipment/supplies (n = 18, 44%), patient safety (n = 14, 34%), and policies/procedures (n = 9, 22%). Work system improvements addressed 27 latent conditions. System changes that eliminated waste or modified the environment to support best practices mitigated 16 latent conditions. System improvements that addressed 44% of LSTs cost the department $11,000 per trauma bay.

**Conclusions:**

TWISST is an innovative and novel strategy that effectively diagnoses and remediates LSTs in a working system. This approach couples highly reliable work system improvements and training into 1 framework.

## INTRODUCTION

Routine safety evaluation is fundamental for safety-critical industries. Health care systems strive to achieve high reliability by identifying errors and near misses. Health care is a complex adaptive system, and human factor-driven behaviors make failure points difficult to distill. Proactively identifying which failure point(s) contribute to catastrophic errors is challenging.^1^ Unsafe work-arounds are clever methods for bypassing work system limitations that impede effective work.^2^ These deviations represent systemic drift from best practices that become normalized over time. They reduce the reliability of the intended work and compromise the quality and safety of care.^2^

Traditional improvement strategies to identify and prevent the recurrence of the same errors are reactive.^1^ Solutions focused on targeting individuals’ knowledge gaps fail to address latent safety threats (LSTs) in the system that are the root of the problem.^3,4^ There is greater buy-in when system improvements are the target of safety initiatives. Understanding resource constraints, elucidating limitations in practical implementation, and integration into the workflow are essential to frontline staff’s successful adoption of system improvements.^5–8^

At our institution, the novel application of simulation augments how we reliably identify LSTs, understand system failures, and eliminate, facilitate, or mitigate errors in our system.^1,7,9,10^ When implementing lean strategies to reduce waste, improve safety, and augment workflow, these initiatives often fail to integrate as intended due to unexpected barriers in local microsystem work culture, workflow, or bedside practice.^11^ Failure to implement sustainable solutions results in wasted resources, recurrent errors, development of workarounds, and drift from best practice.

**Fig. 1.**
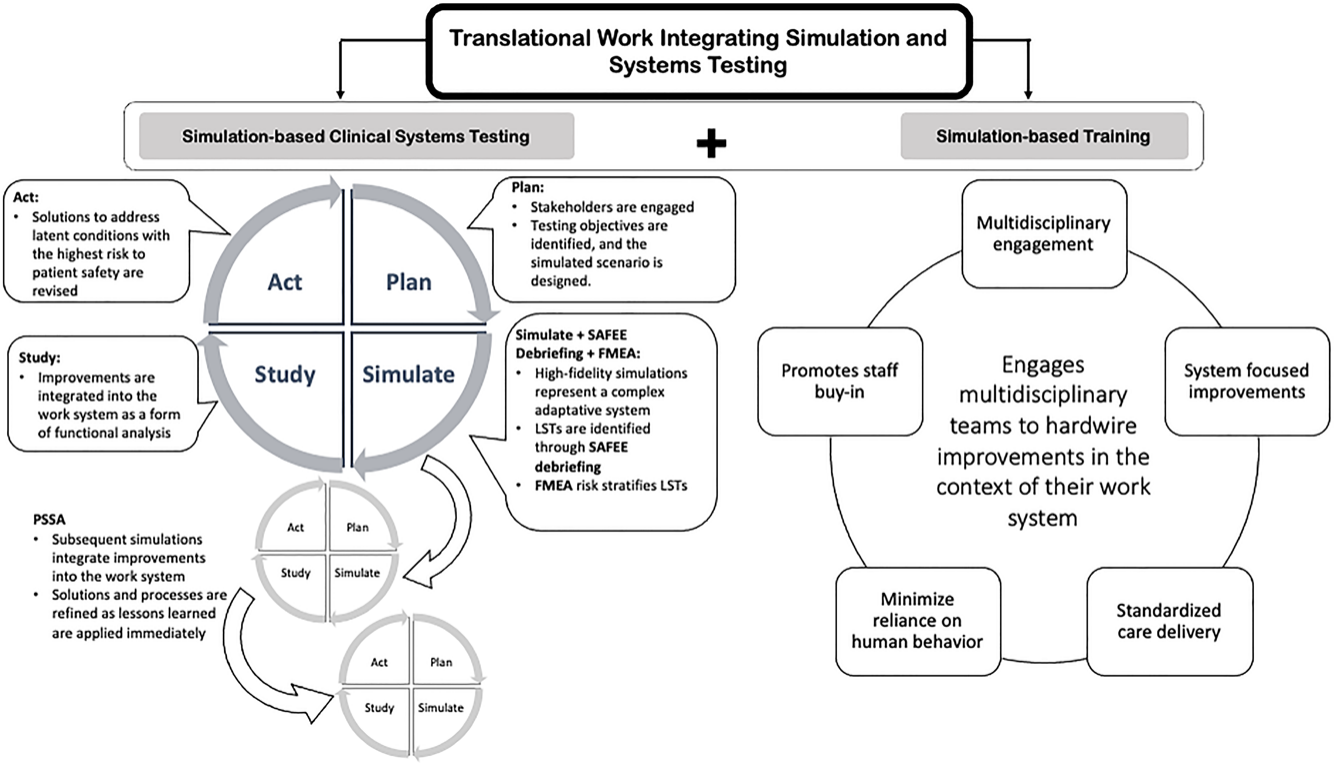
TWISST conceptual model.

Translational Work Integrating Simulation and Systems Testing (TWISST) is a process improvement tool that couples Simulation-based Clinical Systems Testing (SbCST)^12^ with simulation-based training (SbT) (Fig. 1). Like the quality-based method of assessing change, Plan-Simulate-Study-Act (PSSA) is a problem-solving method to identify key drivers for process improvements.^10,13^ This method specifies simulation as the iterative process improvement tool. High-fidelity simulations conducted in TWISST recreate and approximate the realities of complex patient care delivery to demonstrate work-as-done, unpacking the dynamic interaction of people with their work system.^14,15^ Embedding solutions in SbT ensures optimal integration into clinical workflow before widespread implementation, hardwires system improvements, and drives change management.^11^

Here, we present a case study example of TWISST conducted in the Pediatric Emergency Department (PED). In iterative PSSA cycles, frontline teams explored work system and process inefficiencies, identified LSTs, and tested potential solutions. System improvements were hardwired and disseminated through SbT.

## METHODS

SbCST includes simulated scenarios, Summarize, Anchor, Facilitate, Explore, Elicit (SAFEE) debriefing,^16^ and Failure Mode and Effect Analysis (FMEA).^17^ SAFEE debriefing^16^ facilitates the identification of LSTs in work system elements and clinical processes. The SEIPS 2.0 model defines work system elements.^4,18^ The 5 components of the work system include the person (education, skills, and knowledge), organization (teamwork and collaboration), technologies/tools (electronic health record), tasks (cognitive load and job demands), and environment.^4,19^ FMEA risk stratifies the LSTs identified during debriefing.^17^ Stakeholders use the categorized and stratified FMEA report to review LSTs and identify solutions and opportunities for improvement.^20^ SbT applies Rapid Cycle Deliberate Practice and Traditional Reflective Debriefing to embed and hardwire system improvements into practice.

### TWISST; PSSA Cycles

#### Plan:

TWISST took place in the PED between July and November of 2019 and was approved by the hospital’s institutional review board. The simulation guiding team stakeholders included the Pediatric Emergency Medicine (PEM) medical director, 2 PEM attendings, a PEM nursing manager, 2 PEM simulation educators, the respiratory therapist (RT) manager, and 2 institutions’ simulation team members. Stakeholders were engaged, testing objectives were identified, and the simulated scenario was designed during the “plan” phase.

#### Simulate:

In the “simulate” phase, 3 SbCST simulations of a single scenario representing a medical emergency in an *in situ* trauma room in PED identified latent conditions in work system elements defined by SEIPS 2.0. Frontline staff included 2 ER physicians, 3 nurses, 2 RTs, 1 technician, and 1 paramedic. A PediHal S3004, 1-year-old high-fidelity toddler (Gaumard Scientific, Miami, Fla.), a physiologic monitor, and an electronic medical record were used. The mannequin had bag/mask ventilation, intubation, and medication administration capabilities. Staff retrieved disposable supplies or equipment from standard locations as part of their routine workflow.

The charge nurse altered shift team members of a medical emergency, consistent with routine workflow. These staff members made up the ad hoc team participating in SbCST. A prebriefing reviewed goals of the simulation, orientation to the mannequin, and introduction of guiding team members. The single scenario was a patient with hypotensive shock, progressive respiratory failure requiring intubation, and subsequent cardiac arrest. The 20-minute scenario was preprogrammed with a facilitator script detailing vital signs and physical examination findings. Facilitator cues and time-sensitive triggers prompted the progression of each clinical phase, standardizing participant experience and minimizing variation. The same scenario was used for all 3 SbCST simulations. All participants played the role they were accustomed to working. Roles and tasks were assigned based on the team leader’s discretion.

Two members of the simulation team facilitated and codebriefed all 3 scenarios. A structured 35-minute debriefing, using the SAFEE debriefing approach, took place in the trauma room immediately following the scenario. Facilitator-focused debriefing guided participants through the scenario in sequential order of events to maintain situational awareness and engagement. To identify LSTs, the facilitator asked direct questions to assess the accessibility of resources, work efficiency, team performance, ergonomics, and patient and staff safety. The facilitator probed the participants to elicit potential active failures such as barriers and workarounds that contributed to deviations from best practice and process limitations that contributed to drift from optimal performance. Potential active failures included risks to patient safety (poor compliance with standard practice), performance impact (lack of functionality or productivity), employee safety (staff injury), or organizational priorities (regulatory violations).^20^ The facilitator elicited additional feedback to explore potential solutions.

Immediately following each SbCST debriefing, the ED simulation guiding team participated in FMEA scoring using the previously published FMEA scoring process and rubric (**Appendix A, Supplemental Digital Content 1**, http://links.lww.com/PQ9/A495).^11,17,21^

#### Study:

In the “study” phase, subsequent simulations of the same clinical scenario described above integrated system improvements into the workflow as a form of functional analysis. Processes were refined and applied in iterative cycles of improvement. By testing proposed work system changes in each subsequent simulation, barriers to the clinical application of system improvements were quickly identified, and limitations in the clinical application were explored. Furthermore, unsafe downstream consequences were discovered as participants interacted with embedded improvements. Quick refinement and tailoring of complicated processes balanced safety, efficiency, and performance.

#### Act:

In the “act” phase, changes to the system were refined and finalized in preparation for SbT. The guiding team utilized the FMEA report to address LSTs with the highest risk to patient safety. Solutions categorized by work system element integrated lean principles of addressing process, motion, inventory, and waste to target work system gaps and process deficiencies. Guiding team members, PEM administrative, clinical, and operational leaders finalized solutions to LSTs, ensured the feasibility of changes, engaged educators on process changes, and purchased equipment and supplies. In addition, the guiding team finalized the facilitator guides and targeted objectives for SbT. To ensure learners interacted with the changes, the team anchored work system improvements to each phase of the clinical scenario (Table 1).

**Table 1.** Clinical Scenario and Work System Changes

Prebriefing; Familiarization (Orientation to Environment, Equipment, and Available Resources):	
• You will notice there are 2 Broselow carts—one for respiratory and one for nursing. This allows storing more equipment and supplies and enables the RCPs to have their supplies at the head of the bed. • We ask that you open the drawer for your patient’s weight, remove the trays, and bring them to the bedside in this simulation and everyday practice. • You will also notice an IV cart under the table at the med station. • We would like you to use the COW instead of the mounted computer. This is to improve team member positioning. You will hear us talk about the “Docsquad” where the physician and recorder are positioned next to each other. • We have also spent some time reviewing the role of each nurse: we would like to separate the role of the primary nurse from the nurse running or drawing up medications. This is to avoid role hopping and confusion. The bedside nurse who is primary should not leave the patient’s side to leave the room or draw medications. • There is an environmental cue that we would like you to use to help maintain role assignments. A central blue circle is in the tile where the stretcher is positioned. The primary nurse, recorder, physician, and respiratory therapist should remain within the blue circle. In contrast, while runners (paramedics, technicians, and nurses in the “runner” role) should move in and out of the central blue circle.	
**Case Presentation:**A 6-year-old boy with no past medical history. His mother brought him in for vomiting and diarrhoea for 24 hours and then fever starting this morning.	
Scenario	Work System Changes
Phase 1: The nurse brings the patient to the trauma room in a wheelchair	Uses IV cart to obtain access
Phase 2 (5 minutes): Patient develops worsening hypotensive shock requiring fluid resuscitation	Utilizes nursing Broselow cart Uses IV cart to obtain lab draw supplies The D50 in the Broselow cart replaced with D10
Phase 3 (10 minutes): Patient progresses to respiratory failure and requires intubation	Use of medication preparation area Use of workstation on wheels Retrieval of RT Broselow tray for intubation
Phase 4 (5 minutes): Patient develops cardiac arrest on intubation, requiring CPR	Two stools (right and left of the patient) Two backboards (right and left of the patient) Use of workstation on wheels

CPR; cardiopulmonary resuscitation.

### Simulation-based Training

SbT hardwired work system improvements into clinical practice. Twenty-eight SbT workshops were conducted between October and November 2019. Each workshop lasted 3 hours and included the following learners: 1 team lead PEM attending, 1 PEM fellow to assist with intubation, 2–3 nurses, 2 paramedics or medical technicians, and 1 RT. In addition, PEM staff attend as part of their annual competency training. The same preprogrammed medical emergency scenario simulated during SbCST was repeated in the same in situ trauma room in the PED. During the prebriefing, work system changes and process improvements were introduced to the team (Table 1).

A primary facilitator led the debriefing. Directed facilitation in the form of Rapid Cycle Deliberate Practice prompted participants to interact with each work element change as part of the clinical workflow. For example, when paused and coached, team members accessed supplies from the new IV carts, nurse Broselow and RT Broselow carts. Traditional reflective debriefing focused on the rationale behind improvements to level perceptions and suspended assumptions. Learners reflected on their practice and discussed the impact of work system changes on care delivery.

## RESULTS

**Fig. 2.**
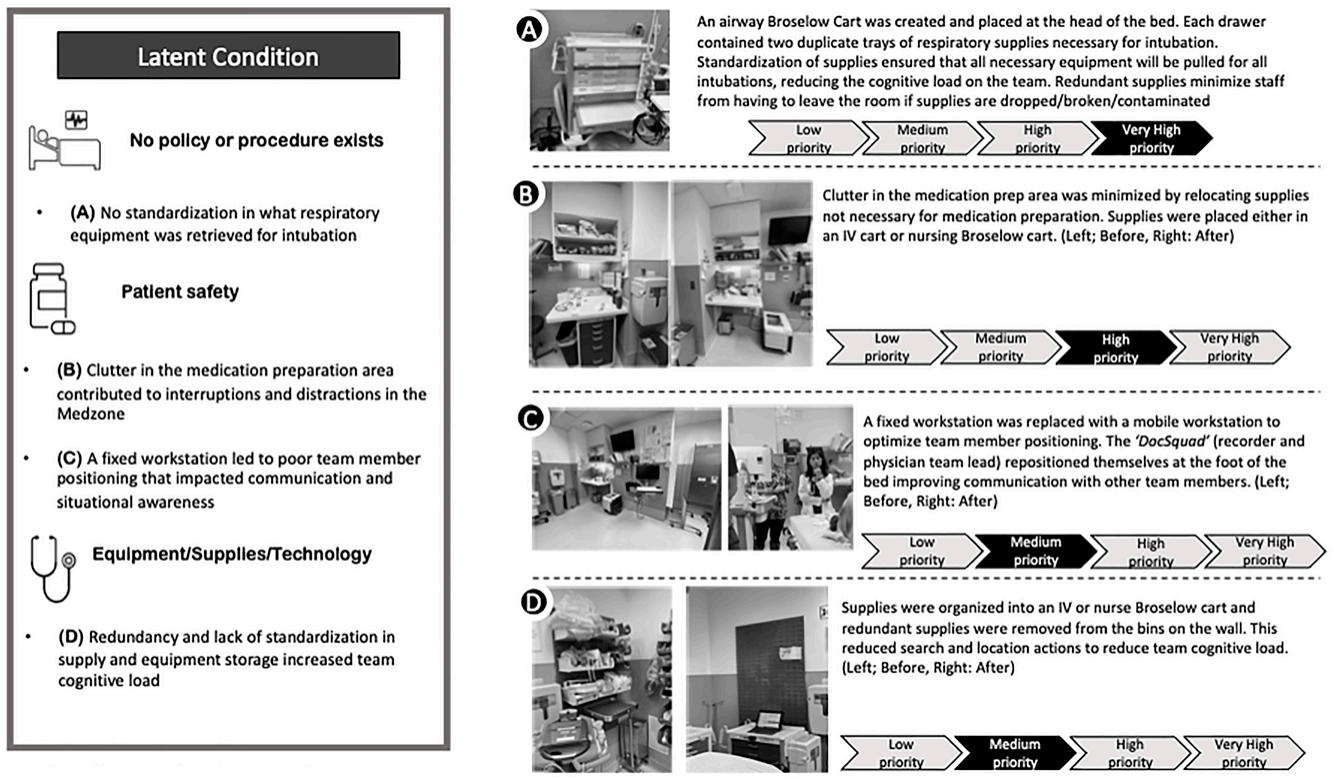
Examples of latent safety threats and solutions.

SbCST identified 41 latent conditions: very high priority (n = 2, 5%), high priority (n = 5, 12%), medium priority (n = 11, 27%), and low priority (n = 23, 56%). These LSTs were also categorized by resource/equipment/supplies issues (n = 18, 44%), gaps in patient safety (n = 14, 34%), and policies/procedures (n = 9, 22%). Work system improvements addressed 27 latent conditions. System changes that eliminated waste or modified the environment mitigated 16 latent conditions. LSTs and solutions categorized by work system element are summarized in Table 2, with examples depicted in Figure 2. The cost associated with improvements is in Table 3.

**Table 2.** Summary of Latent Conditions, Potential Active Failure, Severity, and Proposed Solutions

Work System Category	Latent Safety Threat	Potential Active Failure	Risk Severity	Action Taken
Policies and procedures	No standard practice in terms of what supplies should be retrieved in preparation for intubation	Lack of standardization increases cognitive load Delay in supply retrieval	Very high risk	Implementation of airway Broselow Cart
Team performance	No standard approach to the orientation of team members around the bedside	Negative impact on teamwork and resuscitation ergonomics	Medium priority	Simulation-based intervention learning objective
	The recorder is far away from the physician team leader because the documenting computer is mounted to the wall	Ineffective communication between the team leader and the recording nurse	Medium priority	Implementation of a computer on wheels
	Limited visibility of the monitor for the recording nurse when positioned at the fixed documentation station	Negative impact on resuscitation ergonomics	Low priority	Implementation of a computer on wheels
Resources/supplies/equipment	Lack of available respiratory supplies inside the trauma bay	Delay in supply retrieval Lack of standardization increases cognitive load	High priority	Implementation of airway Broselow cart
	Lack of multiple-size LMAs stocked inside the trauma room	Delay in supply retrieval Lack of standardization increases cognitive load	Medium priority	Implementation of a nurse Broselow cart
	Redundancy in the storage of disposable respiratory supplies	Retrieval of supplies from the medication preparation creates interruptions in the Med-Zone	Medium Priority	Implementation of a nurse Broselow cart
	Redundancy and lack of organization in the storage of disposable nursing supplies	Delay in supply retrieval	Medium priority	Implementation of nursing Broselow cart
	Inaccessibility of laboratory draw supplies, which were stored in the mediation preparation area	Interruptions and disruptions in the Med-Zone	Low priority	Implementation of IV cart
	Inaccessibility of IV supplies	Delay in supply retrieval	Low priority	Implementation of IV cart
	Lack of adequate space at the head of the bed due to team members having to retrieve supplies	Cross-traffic creates disruptions for providers at the head of the bed	Low priority	Implementation of Broselow carts and removal of supplies from the walls
	Inaccessible CPR back board and as a result, staff had to cross the patient’s bedside to retrieve it.	Delay in supply retrieval	Low priority	CPR boards located on the patient’s right and left side of the room
	Lack of D10 in the trauma bay Unused D50 taking up space in the Broselow cart	A cluttered environment creates disruptions and interruptions of the Med Zone	Low priority	The D50 in the Broselow cart replaced with D10
	Lack of tabletop space to prepare medications	A cluttered environment creates disruptions and interruptions in the Med Zone	High priority	The medication preparation area was organized; nonrelevant supplies were removed and placed in carts.

CPR, cardiopulmonary resuscitation; Med Zone, protected area for medication preparation.

**Table 3.** Cost of Changes

Latent Safety Threat	Action Item	Cost (per Single Item)	Number of LSTs Addressed with a Change
Inaccessibility and redundant storage of respiratory supplies	Purchase of a respiratory Broselow cart	$3,330.40	7
Inaccessibility and redundant storage of respiratory supplies	Reorganization of nursing Broselow cart	$0	3
Inaccessibility of IV and lab draw supplies	Purchase of 2 IV carts Decluttering of medication preparation area	$1,810.00	3
Ineffective team member positioning	Purchase of workstation on wheels	$5,849.41	4
Inaccessible back board Inaccessible CPR Stool	Purchase of additional backboard Purchase of additional stool	$100.00 $43.00	1
Total		$11,140.81 (per trauma room)	18

CPR, cardiopulmonary resuscitation.

### Organization

Inconsistent positioning of team members around the bedside during medical emergencies resulted in a lack of adherence to role assignment/role clarity and contributed to role hopping. Nurses switched between being the primary nurse, runner, or medication nurse. Failure to maintain role assignments created workflow disruptions as staff frequently crossed the room as they changed roles. To maintain team member positioning, teams were introduced to an environmental cue, a round blue circle in the tile where the stretcher was positioned. The recorder, physician, primary nurse, and RT were encouraged to remain within the circle while runners moved around the circle’s perimeter, minimizing crowding around the stretcher.

### Tasks

Laboratory draws and intravenous (IV) supplies in the medication preparation area created a cluttered and highly trafficked Med-Zone. Therefore, we removed all nonmedication preparation supplies from the medication area and placed them in 2 carts containing IV start and laboratory draw supplies to minimize interruptions (Fig. 2, example A). In addition, carts to the patient’s right and left were tucked along either side wall when not used. This positioning provided more tabletop surface for medication preparation and minimized cross traffic related to supply retrieval (Fig. 2, example B).

TWISST identified that airway adjuncts (ie, devices used to access and maintain a patent airway) retrieved in preparation for intubation varied based on provider or RT discretion of perceived patient risk factors. Respiratory supply storage was inconsistent and inconvenient, stored in either Broselow carts, wall bins, or automatic dispensing cabinets outside the trauma room. Lack of standardization increased search and locate actions, resulting in inconsistency in supply retrieval. When needed, emergency supplies (laryngeal mask airways, suction, and alternate endotracheal tubes) were not readily available, resulting in delays in care. To reduce search and locate actions, we created a respiratory Broselow cart and placed it at the headwall. Each drawer contained 2 duplicate weight-based trays of respiratory supplies necessary for intubation. Only nonweight-based supplies remained, and all redundant and nonrespiratory supplies were removed from the headwall (Fig. 2, example A).

### Environment

A fixed documentation station mounted at the footwall of the trauma room drove team member positioning. The team leader stood at the footwall close to the recorder. The negative space left between the team leader and the foot of the bed became a footpath for traffic that impeded effective communication. In addition, the recorder and team leader could not maintain global awareness due to poor sightlines to the physiologic monitor and patient, as evidenced by the physician being unaware of changes in the patient’s condition. A workstation on wheels replaced the mounted documentation station. The flexibility allowed the recorder and physician to stand together at the foot of the bed, optimizing resuscitation ergonomics (Fig. 2, example C).

## DISCUSSION

TWISST is an innovative and novel strategy that effectively diagnoses and remediates failure points in a working system. This approach couples highly reliable work system improvements and training into 1 framework. TWISST rectifies system gaps, improves system functioning, and engages multidisciplinary teams to hardwire improvements in the context of their workflow. The PED case example demonstrates how TWISST is uniquely poised to integrate improvement science, safety, systems engineering, and human factors to augment how we discover, investigate, and remediate LSTs.^7^

We are the first to describe a methodology that couples SbCST with SbT. The key element distinguishing TWISST from other simulation initiatives is the application of SbCST methodology in an existing workspace where clinical care was ongoing and pairing it with SbT. In addition, high-fidelity simulations representing a complex adaptative system are necessary to unveil complicated LSTs and break down specific points of failure.

Iterative SbCST-PSSA cycles embedded improvements into the workflow, unveiling barriers to practical application. We remedied obstacles and resolved them before widespread clinical application. Work system-focused solutions that improved frontline work (1) were multifaceted, (2) minimized reliance on education to improve safety, (3) standardized care delivery, (4) optimized the physical environment to support best practice, (5) minimized redundancy, and (6) engaged multidisciplinary teams to calibrate system changes before widespread dissemination. As a result, teams validated that the proposed changes were practical, effectively improved workflow, reduced error-provoking distractions and interruptions, and minimized cognitive load.

Reliance on human behavioral modification to manage risk is ineffective. Therefore, simulation-based education alone is not a comprehensive solution to mitigate challenges related to adherence to best practices.^22,23^ Weaknesses in the system are not addressed if preoccupation with addressing individuals’ knowledge deficits persists. Moreover, the same failures will reoccur if the conditions in which people work remain inadequate.

TWISST identified a lack of adherence to safe medication preparation practices. A simulation-based education initiative on medication safety alone would be time- and resource-consuming. Instead, a simpler solution was to reallocate IV and laboratory supplies. This approach decluttered the workspace and directed traffic away from the area (Fig. 2, example B). Similarly, the mounted computer drove team member positioning. A workstation on wheels provided the flexibility that optimized team member positioning. Positioning the physician and recorder at the foot of the bed (the *“docsquad,”* Fig. 2, example C) created a triangle between the medication delivery nurse, the recorder, and the team leader, facilitating communication, especially during medication administration (Fig. 2, example C).

A central blue circle in the tile under the patient stretcher was an environmental cue that augmented team performance by helping members maintain role assignments. When brought to the teams’ attention, this simple environmental cue became an intuitive marker that minimized role hopping. During SbT, team members reminded each other to “stay within the circle,” reinforcing the maintenance of role assignments.

The creation of the airway Broselow cart minimized subjectivity and variability in decision-making. It improved supply accessibility to optimize workflow efficiency (Fig. 2, example A). Staff intuitively obtained supplies from the conveniently placed RT Broselow cart. Without major modifications to clinical practice, such as implementing a checklist, supply standardization ensured that the team retrieved the same airway supplies for all intubations irrespective of patient condition or provider decision-making. Optimized space utilization improved familiarity and predictability with the space and reduced wasted motion and seeking/searching actions contributing to errors.^24,25^

Renovations and retrofitting an existing clinical space are often cost prohibitive, so changes to the built environment are overlooked and underemphasized.^21,26^ TWISST clarified how the interaction of the physical environment and human-driven behaviors contributed to LSTs. Improvements focused on the physical environment (a work system element) influenced human behavior and mitigated LSTs without relying on educational training and human behavioral modification. Moreover, we demonstrated that environmental optimization could be done relatively cheaply. For example, system improvements that addressed 44% of LSTs cost the PEM department $11,000 per trauma bay.

TWISST served as a catalyst to transform perspective. Teams saw care delivery through a bird’s eye view that looked at their whole work system instead of focusing on individual performance gaps. Barriers in their work environment that inhibited efficient and safe care delivery became opportunities for improvement instead of challenges to work around. This empowered teams to engage in the discovery of system gaps and inefficiencies. Frontline staff became partners in problem-solving, and leaders did not enforce changes in a top-down approach. This sense of ownership and responsibility fosters resilience. Implementation of TWISST shifted the reactive approach to systems probing to a proactive approach that rose to the challenge of implementing impactful and effective solutions with input from frontline teams.

### Challenges and Limitations

Project success relies on invested stakeholders engaged in the simulation testing process and FMEA scoring and who are accountable for devising and implementing solutions. High-fidelity simulations that approximate reality and represent complex adaptive systems are necessary to detect and unpack complex LSTs and failure points. Low-fidelity simulations are more reflective of imagined work rather than work as done. The former requires more simulation resources, which may be challenging for some centers. While our specific LSTs may not be generalizable to other centers, TWISST applies to any clinical space or patient population.

This study lacks ecological validity as the LSTs identified were subjective and rooted in the microsystem’s workflow, culture, and the frontline staff’s prior experience and perceptions. Furthermore, we did not have access to immediate human factor expertise; therefore, no human factors analysis was available to validate LSTs or implemented solutions.

## CONCLUSIONS

High-reliability systems will never reach zero harm; therefore, the cycle of discovery and improvement remains an endlessly iterative process. Like pluralistic walkthroughs, common usability testing, and user-centered design, TWISST focused on observing, understanding, and evaluating clinicians and their complex interaction with their work environment. TWISST integrates improvement science, patient safety, systems engineering, and human factors to unpack pieces of a complex adaptive system, improve performance, mitigate risk, promote reliability, and reduce human errors.

## ACKNOWLEDGMENT

Assistance with the study: We acknowledge the Pediatric Emergency Department’s Simulation Guiding Team for contributing to this work.

## APPENDIX A

Failure Mode and Effect Analysis rubric for scoring of latent safety threats

## DISCLOSURE

The authors have no financial interest to declare in relation to the content of this article.

## Supplementary Material
