# Supplementary material for: Let us to the TWISST; Plan, Simulate, Study and Act
Source: Pediatr Qual Saf. 2023 Jul 10;8(4):e664. doi: 10.1097/pq9.0000000000000664 (PMC10332833; doi:10.1097/pq9.0000000000000664)
Supplement: Supplementary file 1 [file pqs-8-e664-s001.pdf]

FAILURE MODES AND EFFECTS ANALYSIS (FMEA) SCORING TOOL

Risk Priority Number** is calculated by multiplying **Severity*Detection* Occurrence

Green = Low priority (1-27), Yellow = Medium priority (28-63), High priority (64-99), Very high priority (100-125)

	5	4	3	2	1
**SEVERITY**	**CATASTROPHIC**	**MAJOR**	**MODERATE**	**MINOR**	**NO HARM**
	**Patient Safety** [a]<br>- Failure mode could result in permanent patient harm or death.	**Patient Safety**<br>- Failure mode could result in initial or prolonged hospitalization and cause temporary patient harm.	**Patient Safety**<br>- Failure mode could result in the need for increased patient monitoring, treatment, and/or in intervention, but there is no patient harm.	**Patient Safety**<br>- Failure mode reached patient but caused no harm.	**Patient Safety**<br>- Failure mode did not reach the patient.
	**Staff Safety**<br>- Failure mode results in loss of work for >90 days. (Long-term disability)	**Staff Safety**<br>-Failure mode results in loss of work for >8 days up to 90 days. (Short term disability)	**Staff Safety**<br>-Failure mode results in loss of work for less than 8 days.	**Staff Safety**<br>-Failure mode results in missed work time during same shift.	**Staff Safety**<br>-No loss of work
	**Policies and Procedures**<br>- No policy/procedure is in place<br>- Workflow does not support safe patient care	**Policies and Procedures**<br>- Policy/procedure is in place, but needs to be modified<br>- Workarounds compromise clinical activities	**Policies and Procedures**<br>- Policy/procedure is in place, but was not followed<br>- Workarounds created to optimize workflow do not align with best practices	**Policies and Procedures**<br>- Policy/procedure is in place, but staff is unaware<br>- Workarounds have been created to optimize workflow	**Policies and Procedures**<br>- Policy/procedure is in place<br>- Workflow supports safe patient care
	**Equipment/Supplies/Technology** [b]<br>- Staff experiences lack of functionality.<br>- Item completely fails to meet intended needs and performance is completely lost.<br>- Equipment/supplies/technology not available at all	**Equipment/Supplies/Technology**<br>- Staff experiences a reduction in performance and productivity.<br>- Failure can be overcome with modification, but there is some performance loss.<br>- Equipment/supplies/technology available but does not function at all/retrieval delays care	**Equipment/Supplies/Technology**<br>- Staff experiences a reduction in convenience.<br>- Failure can be overcome with modification, but there is no performance loss.<br>- Equipment/supplies/technology available but does not function as intended or location is inconvenient	**Equipment/Supplies/Technology**<br>- Staff experiences annoyance.<br>- Failure can be overcome without modification or performance loss.<br>- Equipment/supplies/technology available but staff does not know how to use or access it	**Equipment/Supplies/Technology**<br>- Failure mode goes unnoticed by staff.<br>- No need for modification, no loss in performance.<br>- Equipment/supplies/technology available and staff know how to use it
	**Patient and Family Experience** [e]<br>- Any grievance that requires referral to CMS.	**Patient and Family Experience**<br>- Any grievance that requires involvement of Patient Rep or review by Patient Safety and Risk Management.	**Patient and Family Experience**<br>- Any complaint or issue expressed to management that can be resolved promptly.	**Patient and Family Experience**<br>- Any complaint or issue expressed verbally to staff that can be resolved promptly.	**Patient and Family Experience**<br>- No complaints or issues expressed.
	**Cost to the Organization** [f]<br>- ≥$250,000<br>**Accreditation/regulatory finding** [g]<br>-Immediate Jeopardy	**Cost to the Organization:**<br>-$100,000-$250,000<br>**Accreditation/regulatory finding**<br>-Condition	**Cost to the Organization**<br>- $10,000-$100,000<br>**Accreditation/regulatory finding**<br>-Standard	**Cost to the Organization:**<br>-<$10,000<br>**Accreditation/regulatory finding**<br>-None	**Cost to the Organization:**<br>-None<br>**Accreditation/regulatory finding**<br>-None

**OCCURRENCE**	**FREQUENT**<br>Likely to occur more than once in a 24-hour period	**OFTEN**<br>Probably will occur once a day	**SOMETIMES**<br>Possible to occur weekly	**OCCASIONALLY**<br>Possible to occur monthly	**SELDOM**<br>Unlikely to occur in a 6-month period
**DETECTION**	**ALMOST IMPOSSIBLE**<br>No known controls are available to detect failure mode	**REMOTE**<br>Remote likelihood that current controls will detect failure mode	**MODERATE**<br>Moderate likelihood that current controls will detect failure mode	**HIGH**<br>High likelihood that current controls will detect failure mode. Reliable detection controls are known with similar processes	**VERY HIGH**<br>Current controls will detect failure mode. Reliable detection controls are known for this particular process

Appendix A; Failure Mode and Effect Analysis Scoring Rubric
